# Supplementary material for: muscat detects subpopulation-specific state transitions from multi-sample multi-condition single-cell transcriptomics data
Source: Nat Commun. 2020 Nov 30;11:6077. doi: 10.1038/s41467-020-19894-4 (PMC7705760; doi:10.1038/s41467-020-19894-4)
Supplement: Supplementary file 1 — Supplementary Information [file 41467_2020_19894_MOESM1_ESM.pdf]

Supplementary material:
muscat detects subpopulation-specific state transitions from multi-sample multi-condition single-cell transcriptomics data

Helena L. Crowell[1,2], Charlotte Soneson[1,2,3,*], Pierre-Luc Germain[1,4,*], Daniela Calini[5], Ludovic Collin[5], Catarina Raposo[5], Dheeraj Malhotra[5] & Mark D. Robinson[1,2]

[1] *Department of Molecular Life Sciences, University of Zurich, Zurich, Switzerland*

[2] *SIB Swiss Institute of Bioinformatics, Zurich, Switzerland*

[3] *Present address: Friedrich Miescher Institute for Biomedical Research and SIB Swiss Institute of Bioinformatics, Basel, Switzerland*

[4] *D-HEST Institute for Neuroscience, Swiss Federal Institute of Technology, Zurich, Switzerland*

[5] *F. Hoffmann-La Roche Ltd, Pharma Research and Early Development, Neuroscience, Ophthalmology and Rare Diseases, Roche Innovation Center Basel, Basel, Switzerland*

[*] *These authors contributed equally.*

Method ID	Input data	Agg.	Model level	Ref.
edgeR.sum(counts)	counts	✓	cluster-sample PBs	Robinson et al.[1]
edgeR.sum(scalecpm)	LS scaled pseudobulk CPM	✓	cluster-sample PBs	
limma-voom.sum(counts)	counts	✓	cluster-sample PBs	Ritchie et al.[2]
limma-trend.mean(logcounts)	$\log_2$ LS normalized counts	✓	cluster-sample PBs	
limma-trend.mean(vstresiduals)	VST residuals	✓	cluster-sample PBs	
MM-dream	counts	✗	SCs; cluster-level	Hoffman and Schadt[3]
MM-dream2	counts	✗	SCs; cluster-level	Hoffman and Roussos[4]
MM-nbinom	counts	✗	SCs; cluster-level	
MM-vst	VST residuals	✗	SCs; cluster-level	
scDD.logcounts	$\log_2$ LS normalized counts	✗	SCs; cluster-level	Korthauer et al.[5]
scDD.vstresiduals	VST residuals	✗	SCs; cluster-level	
MAST.logcounts	$\log_2$ LS normalized counts	✗	SCs; cluster-level	Finak et al.[6]
AD-gid.logcounts	$\log_2$ LS normalized counts	✗	SCs; cluster-group level	Scholz and Stephens[7]
AD-gid.vstresiduals	VST residuals	✗	SCs; cluster-group level	
AD-sid.logcounts	$\log_2$ LS normalized counts	✗	SCs; cluster-sample level	
AD-sid.vstresiduals	VST residuals	✗	SCs; cluster-sample level	

Supplementary Table 1: Overview of compared DS analysis methods. From left to right: Method identifier as depicted in all figures; input data; whether data is aggregated or not; the levels at which differential testing is performed; reference. (Agg. = aggregation, CPM = counts per million, LS = library size, VST = variance stabilizing transformation, PBs = pseudobulks, SCs = single cells)

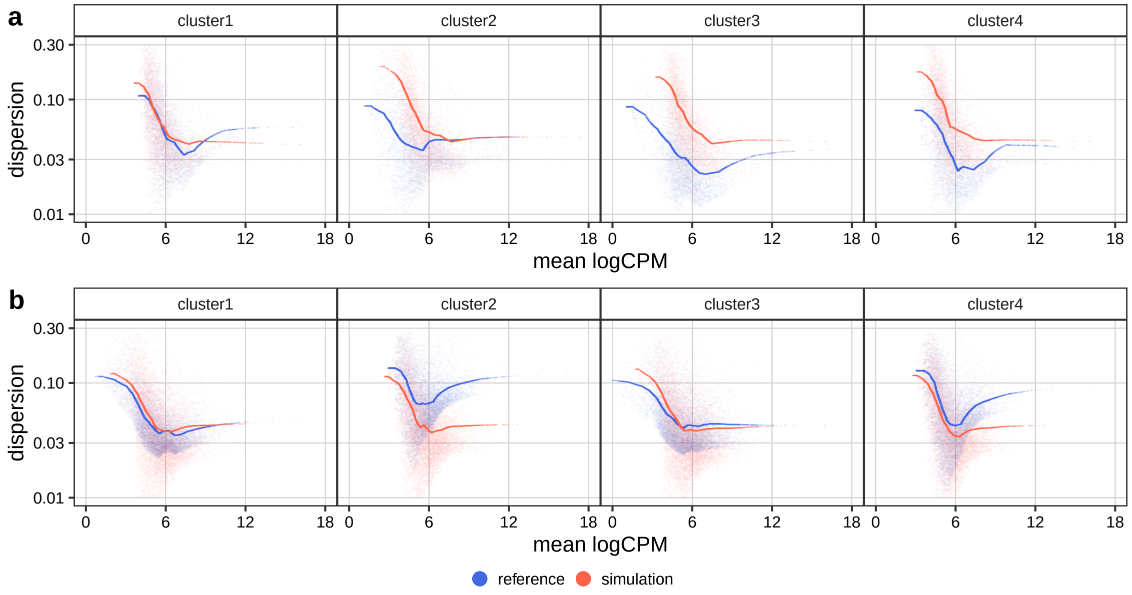

Supplementary Figure 1: Comparison of pseudobulk-level mean-dispersion estimates for reference vs. simulated data, separated by subpopulation. Lines correspond to trended dispersion estimates; faded points represent tag-wise dispersion estimates. Lower (1%) and upper (99%) dispersion quantiles were removed for visualization. Simulations are based on the Kang et al.[8] (**a**) and LPS dataset (**b**) as reference, respectively.

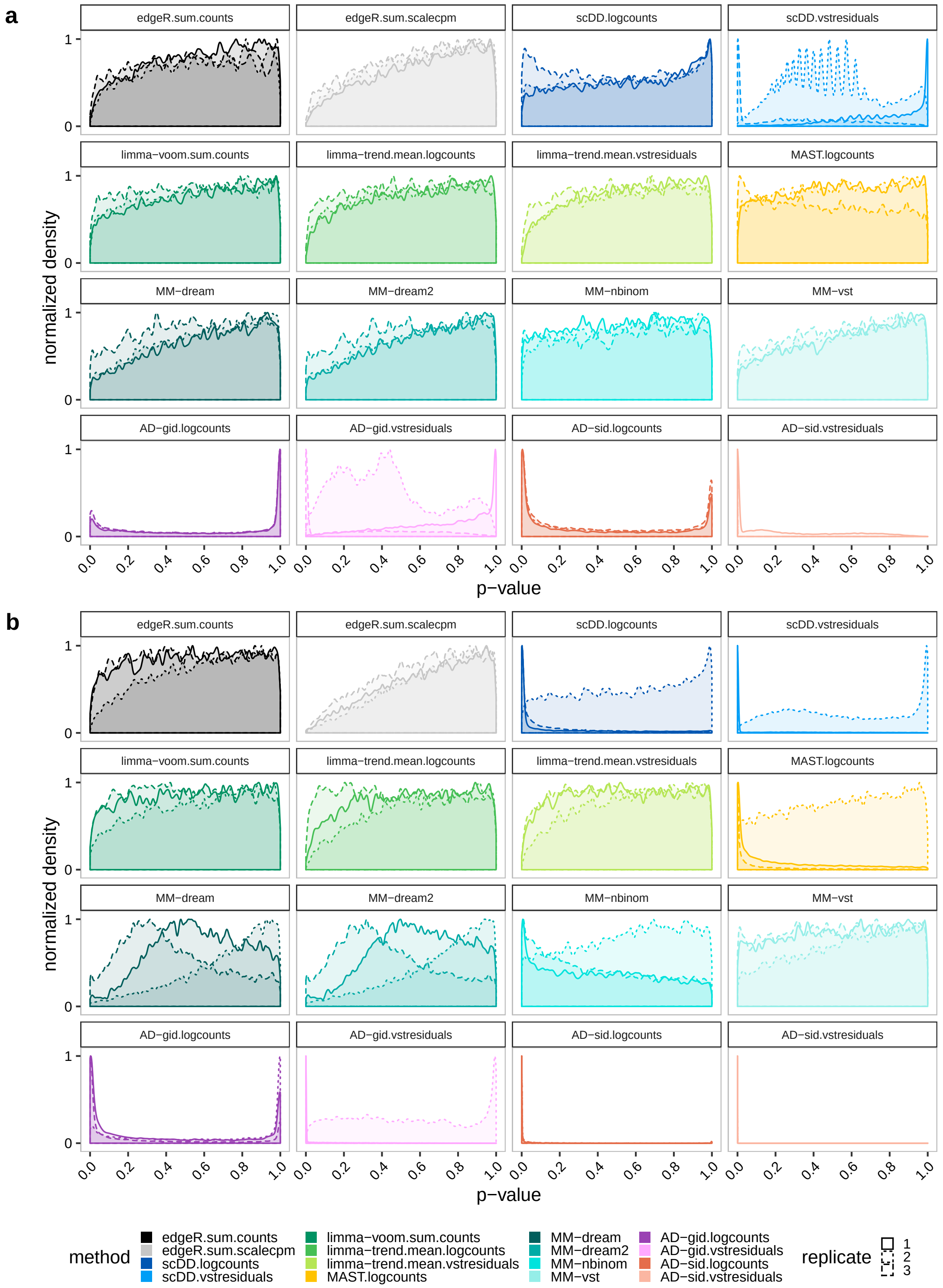

Supplementary Figure 2: Nominal p-value distributions (densities) obtained from three null simulation replicates, stratified by method. Each simulation run includes 3 samples per group, and 2000 genes tested across 2 clusters; 3 simulation runs are shown. Densities that are near-uniform are consistent with data lacking differential signal. Simulations are based on the Kang et al.[8] (**a**) and LPS dataset (**b**) as reference, respectively.

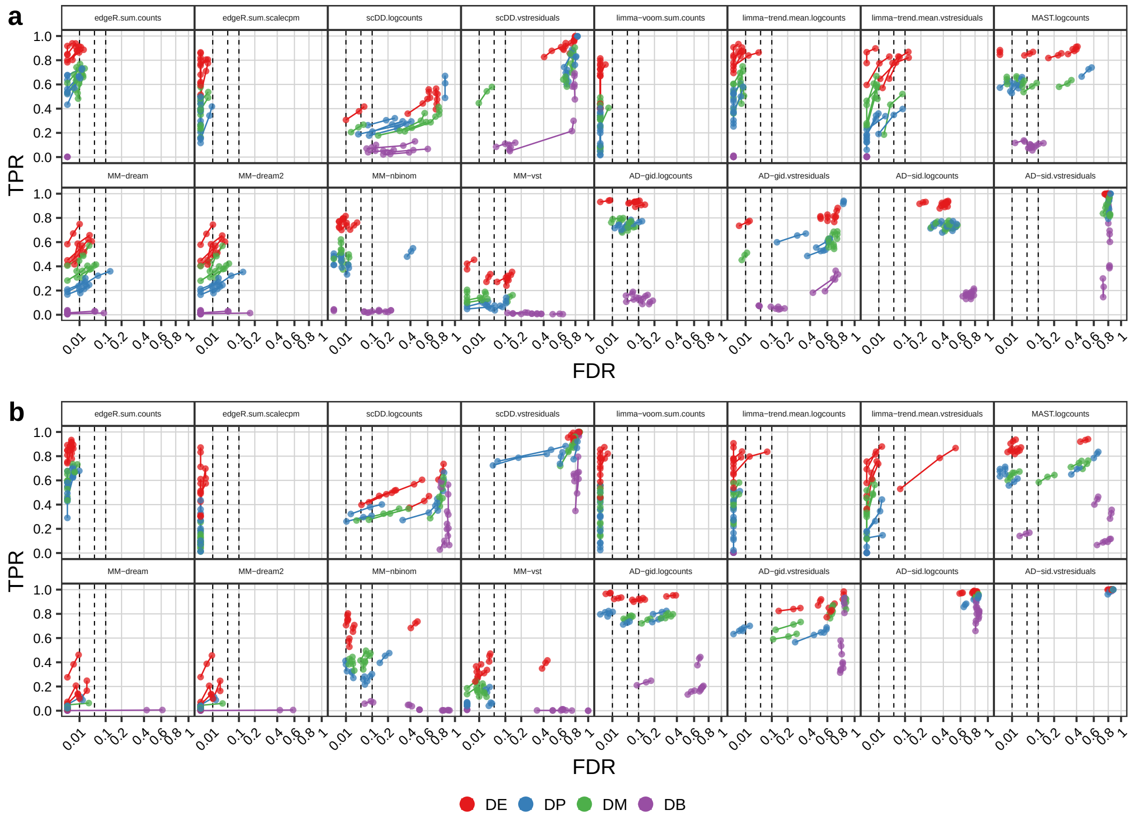

Supplementary Figure 3: DS method performances across differential distribution and simulation replicates. Points correspond to observed overall true positive rate (TPR) and false discovery rate (FDR) values at FDR cutoffs of 1%, 5%, and 10%; dashed lines indicate desired FDRs. Each group of inter-connected points corresponds to one simulation with 10% of DS genes (of the type indicated by their color). Simulations are based on the Kang et al.[8] (**a**) and LPS dataset (**b**) as reference, respectively.

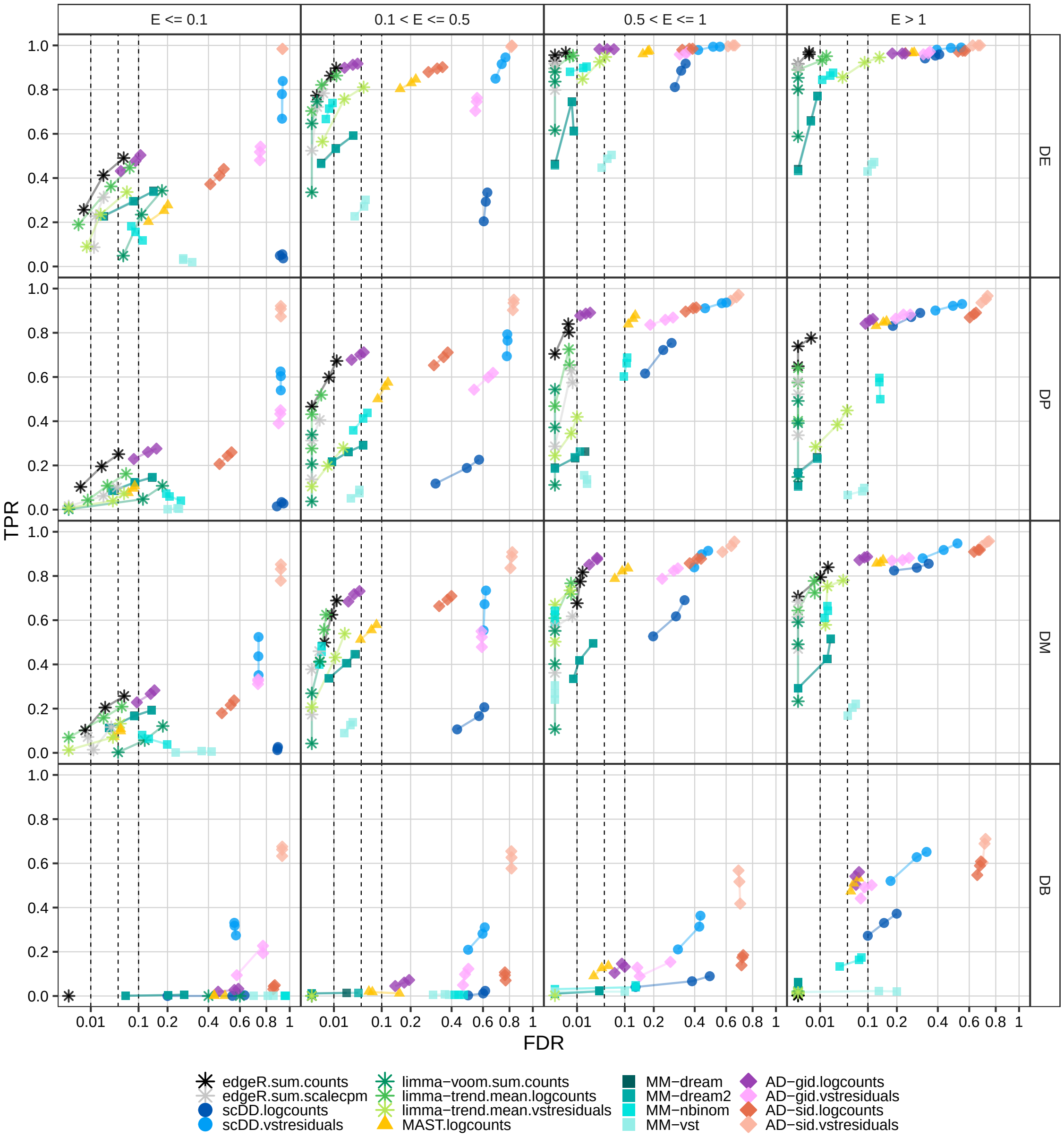

Supplementary Figure 4: DS method performances across expression-levels and differential distribution categories; Kang et al.[8] dataset reference. Points correspond to observed overall true positive rate (TPR) and false discovery rate (FDR) values at FDR cutoffs of 1%, 5%, and 10%; dashed lines indicate desired FDRs. Results were stratified into groups according to the mean of simulated expression-means across groups. For each panel, performances were averaged across 5 simulation replicates, each containing 10% of DS genes.

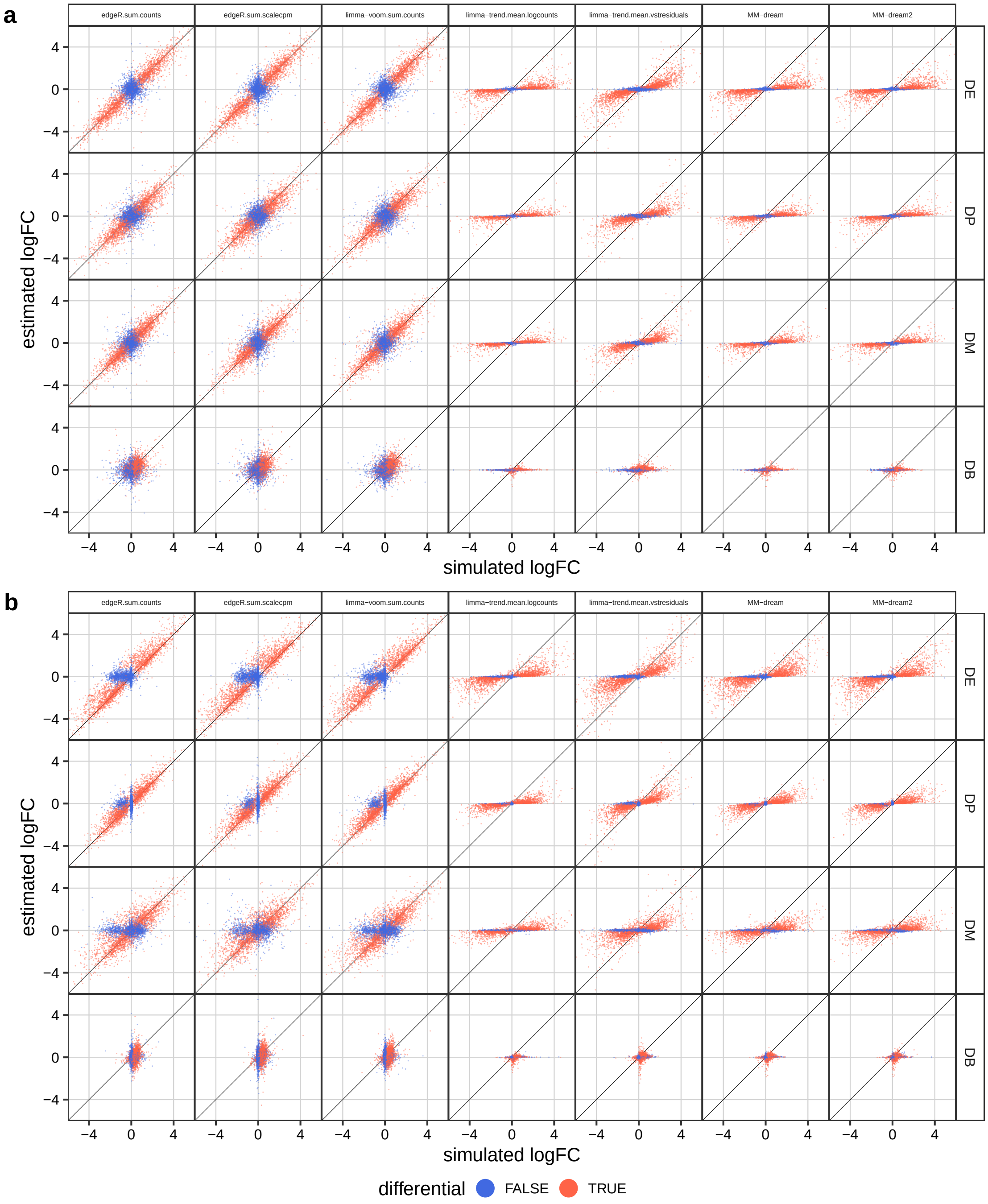

Supplementary Figure 5: Simulated vs. estimated cross-group log-fold changes (logFC), stratified by method and gene category. Each point corresponds to a gene-subpopulation instance; coloring corresponds to non-differential (blue) or truly differential (red). Included are only methods that return logFC estimates. For plotting, a random subset of 2'000 points was sampled per method, simulation, and color. Simulations are based on the Kang et al.[8] (**a**) and LPS dataset (**b**) as reference, respectively.

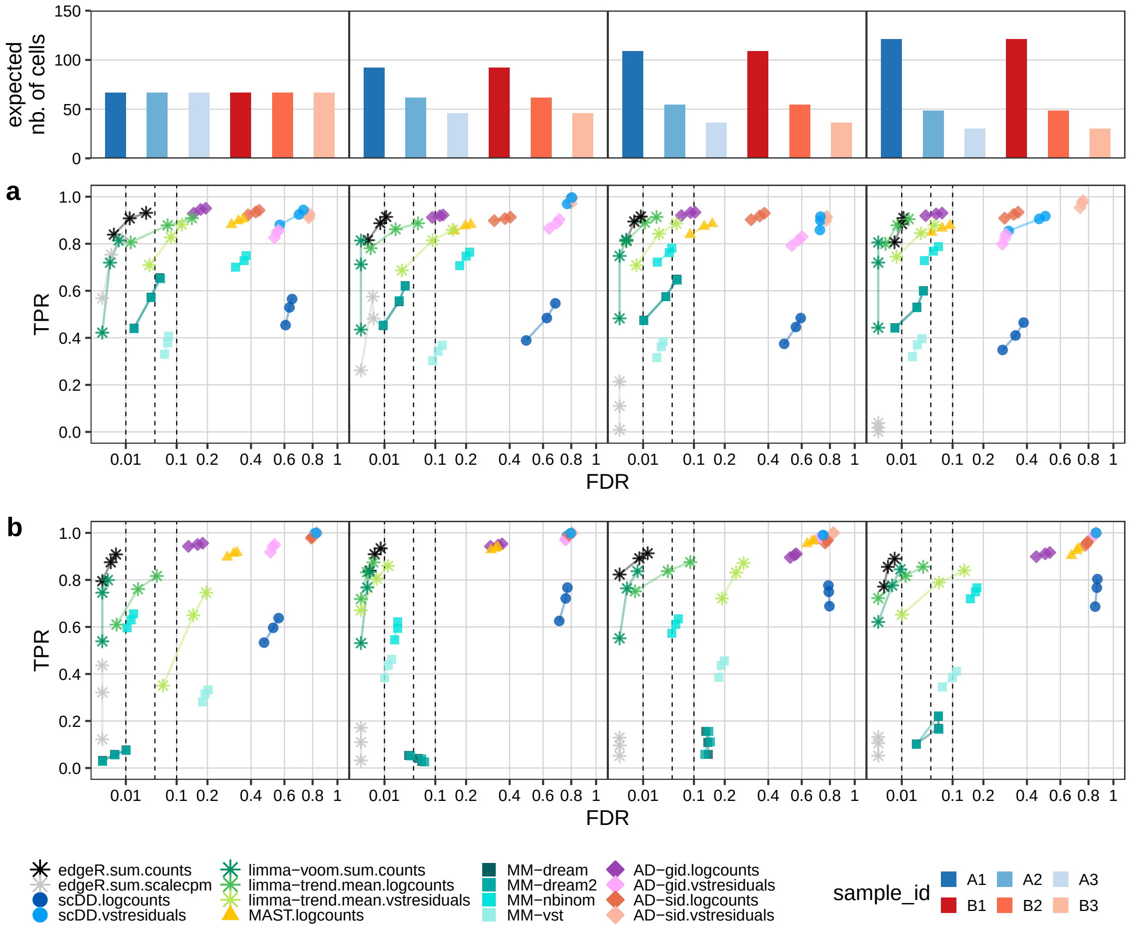

Supplementary Figure 6: Effects of unbalanced sample sizes on DS method performances. Points correspond to observed overall true positive rate (TPR) and false discovery rate (FDR) values at FDR cutoffs of 1%, 5%, and 10%; dashed lines indicate desired FDRs. Results were stratified into groups according to the variance of simulated sample sizes. For each panel, performances were averaged across 5 simulation replicates, each containing 10% of DS genes. Simulations are based on the Kang et al.[8] (**a**) and LPS dataset (**b**) as reference, respectively.

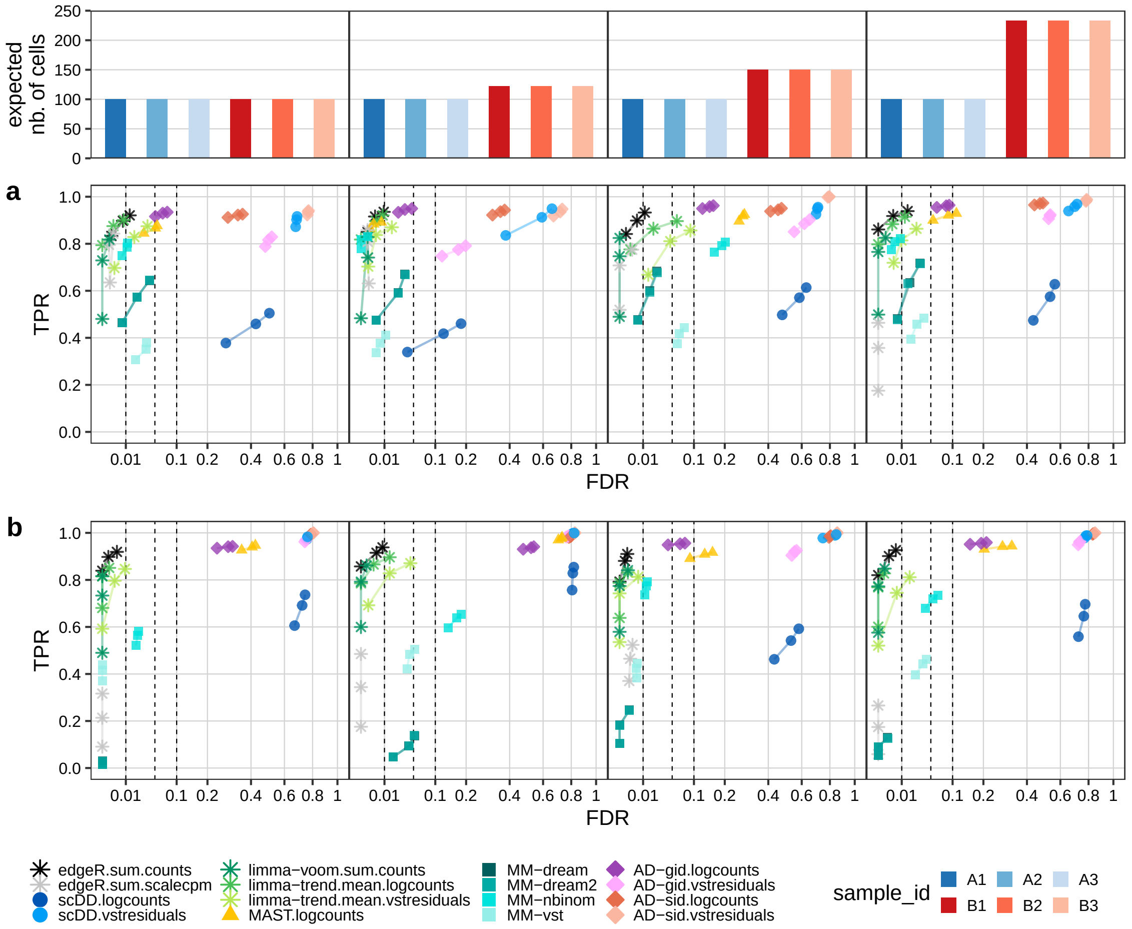

Supplementary Figure 7: Effects of unbalanced group sizes on DS method performances. Points correspond to observed overall true positive rate (TPR) and false discovery rate (FDR) values at FDR cutoffs of 1%, 5%, and 10%; dashed lines indicate desired FDRs. Results were stratified into groups according to the variance of simulated group sizes. For each panel, performances were averaged across 5 simulation replicates, each containing 10% of DS genes. Simulations are based on the Kang et al.[8] (**a**) and LPS dataset (**b**) as reference, respectively.

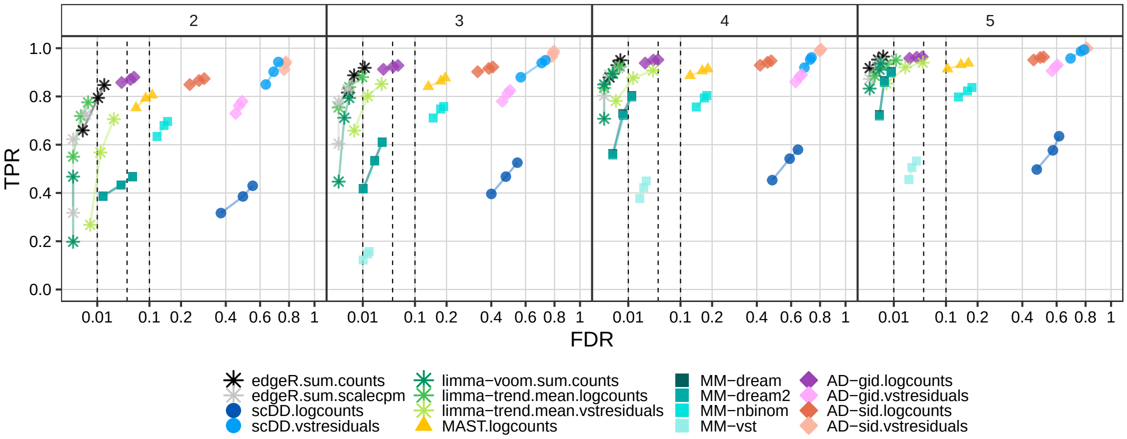

Supplementary Figure 8: Effect of the number of replicates per group on DS method performances; Kang et al.[8] dataset reference. Points correspond to observed overall true positive rate (TPR) and false discovery rate (FDR) values at FDR cutoffs of 1%, 5%, and 10%; dashed lines indicate desired FDRs. Results were stratified into groups according to the number replicates in each group. For each panel, performances were averaged across 5 simulation replicates, each containing 10% of DS genes.

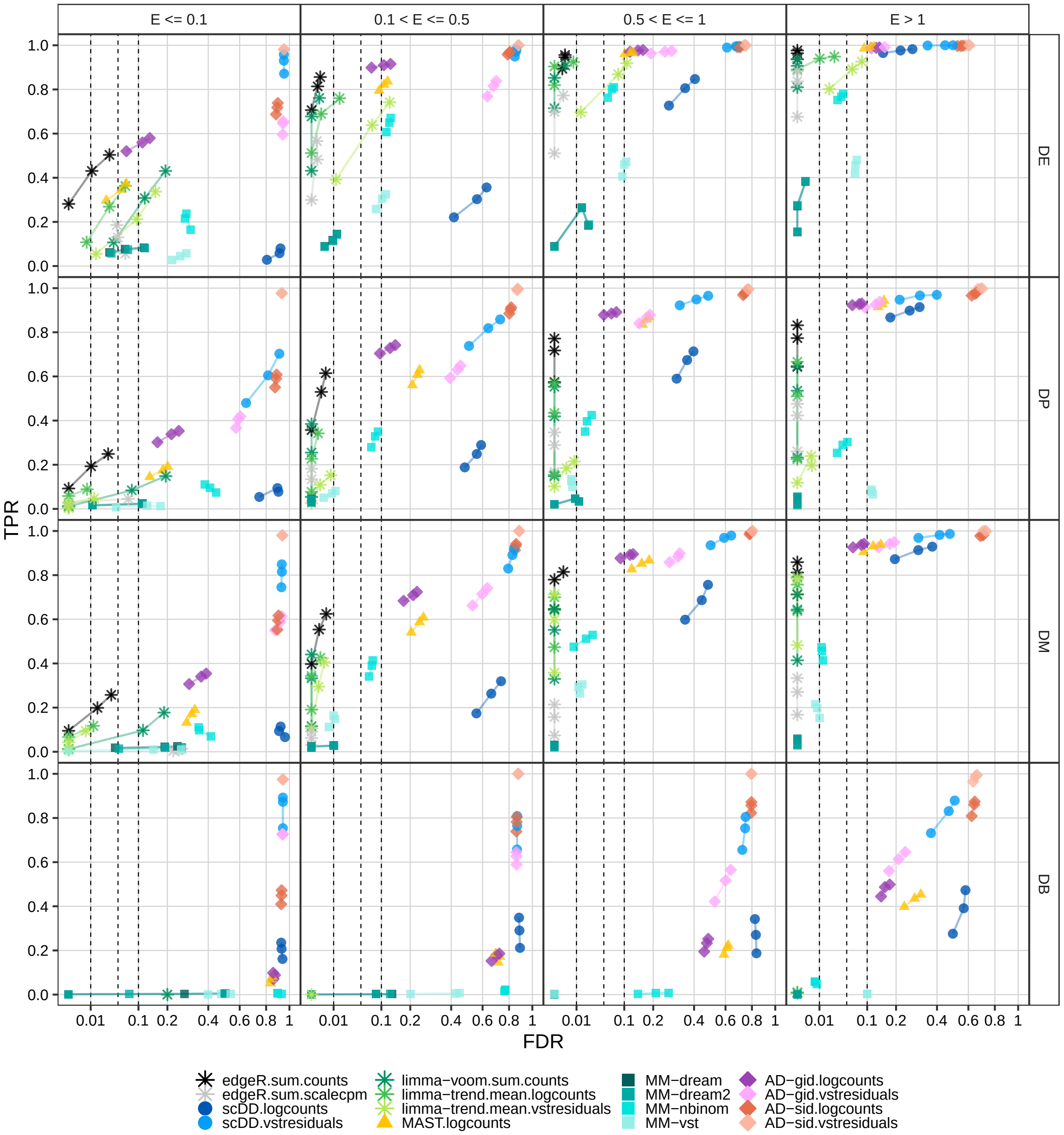

Supplementary Figure 9: DS method performances across expression levels and differential distribution categories; LPS dataset reference. Points correspond to observed overall true positive rate (TPR) and false discovery rate (FDR) values at FDR cutoffs of 1%, 5%, and 10%; dashed lines indicate desired FDRs. Results were stratified into groups according to the mean of simulated expression-means across groups. For each panel, performances were averaged across 5 simulation replicates, each containing 10% of DS genes (of the type specified in the right-hand side panel labels).

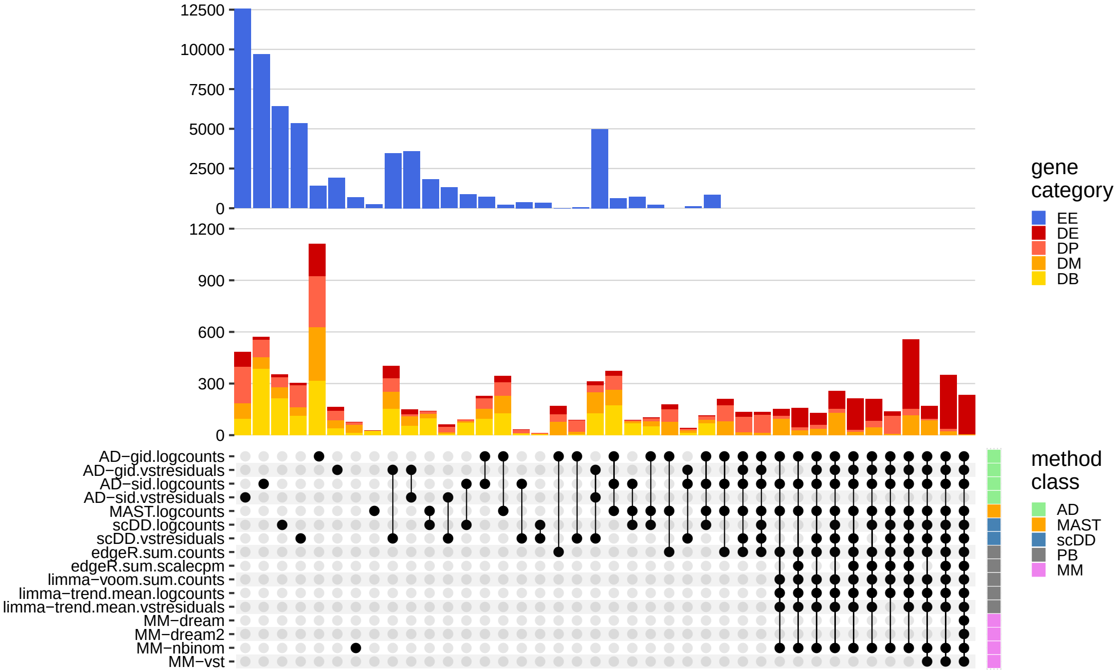

Supplementary Figure 10: Between-method concordance; LPS dataset reference. Upset plot obtained from intersecting the top-n ranked differential genes, where $n = min(n_1, n_2)$, where n_1 = number of genes simulated to be differential, and n_2 = number of genes called differential at FDR < 0.05. Shown are the 40 most frequent interactions; coloring corresponds to (true) simulated gene categories.

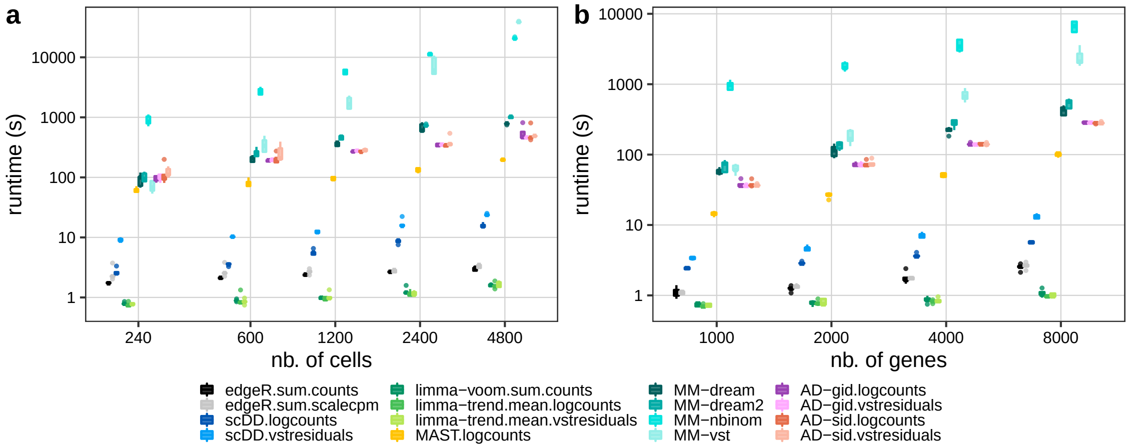

Supplementary Figure 11: DS method runtimes vs. number of cells (**a**) and number of genes (**b**). Included are runtimes from 5 simulation replicates per subset of cells and genes, respectively, using the Kang et al.[8] dataset reference; single-core computing times were recorded.

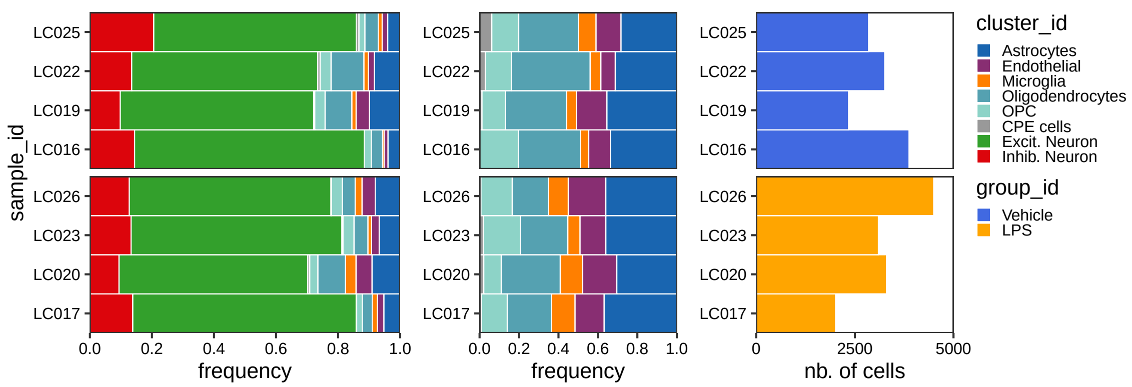

Supplementary Figure 12: Relative and absolute subpopulation abundances for the LPS dataset. The left panel shows sample-wise frequencies of the annotated subpopulations; the middle panel shows relative frequencies after removal of all neuronal subpopulations; the right panel shows the number of cells per sample after filtering.

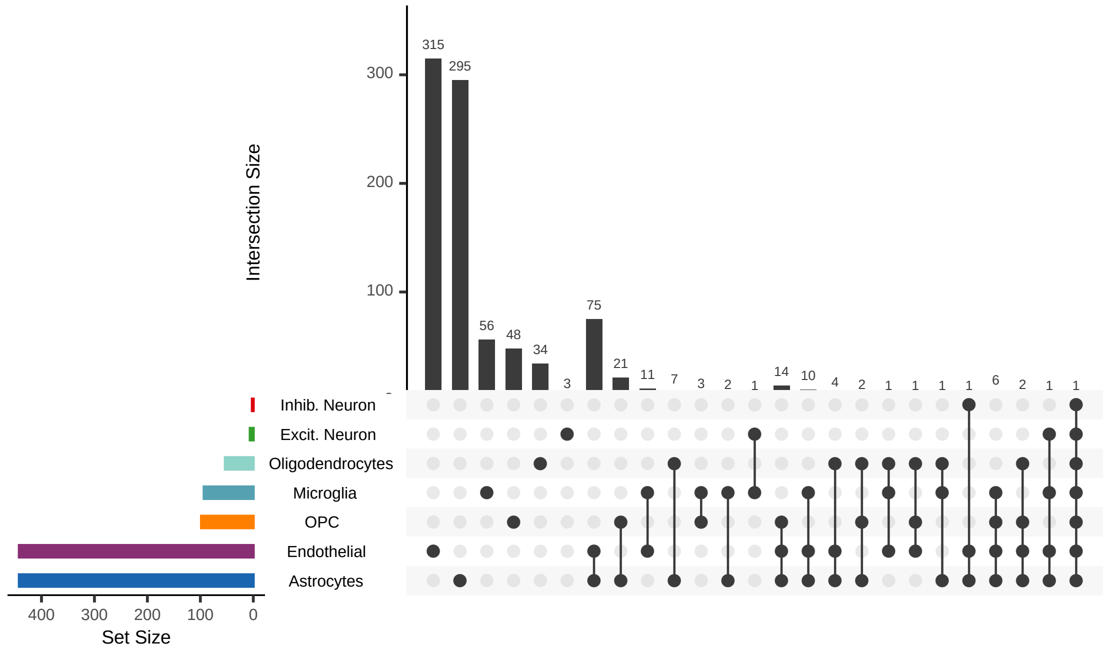

Supplementary Figure 13: Upset plot of differentially expressed genes identified for the LPS dataset, by detected subpopulation. Included are genes with FDR < 0.05 and $|\text{logFC}| > 1$; shown are all subpopulations intersections with non-zero size.

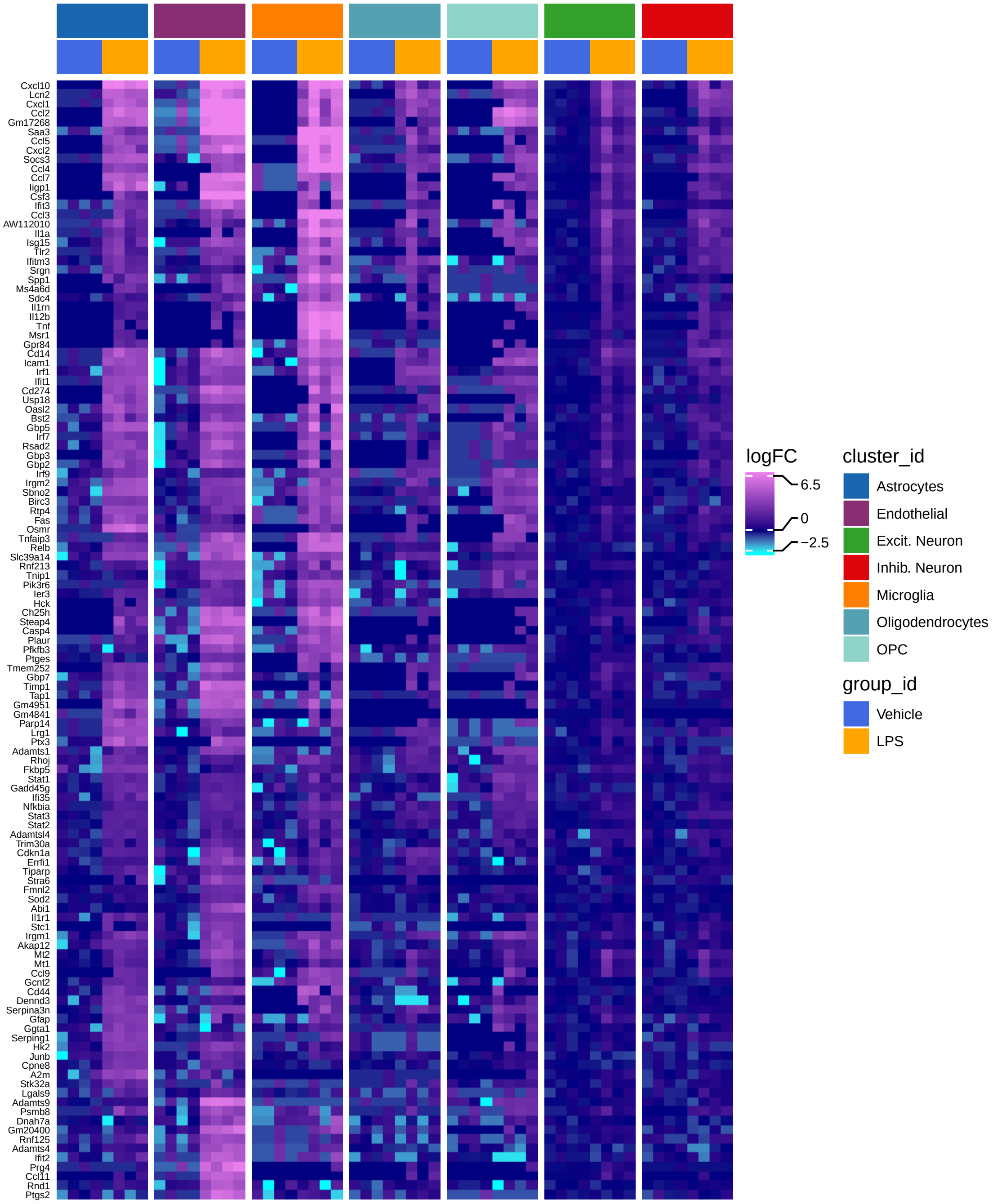

Supplementary Figure 14: Heatmap of cross-group logFCs of DE genes with consensus cluster ID 3 for the LPS dataset. Included are DE genes with FDR $< 1\text{e}{-4}$ and $|\text{logFC}| > 1$. For every gene, the displayed log-fold-change (logFC) is normalized to that gene's average expression in the vehicle group (in the corresponding subpopulation); top and bottom 1% logFC quantiles were truncated for visualization.

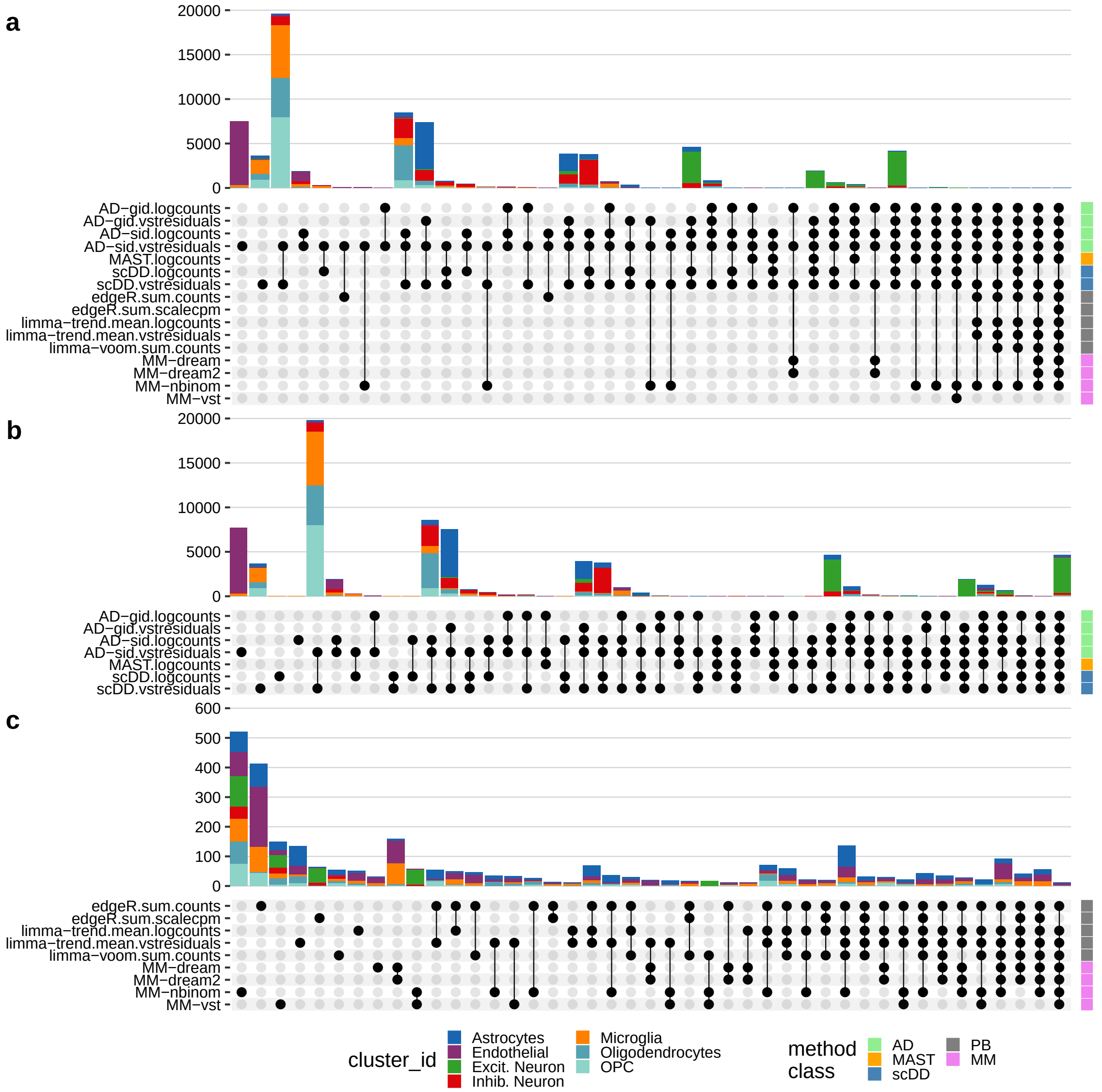

Supplementary Figure 15: Upset plot of differential state genes detected for the LPS dataset, by method and across all subpopulations (excluding CPE cells). Included are genes with FDR < 0.05; shown are the 40 most frequent intersections between all methods (**a**), AD, MAST and scDD methods (**b**), and aggregation- and MM-based methods (**c**).

References

[1] Mark D Robinson, Davis J McCarthy, and Gordon K Smyth. edgeR: a Bioconductor package for differential expression analysis of digital gene expression data. *Bioinformatics*, 26(1):139–140, 2010.

[2] Matthew E Ritchie, Belinda Phipson, Di Wu, Yifang Hu, Charity W Law, Wei Shi, and Gordon K Smyth. limma powers differential expression analyses for RNA-sequencing and microarray studies. *Nucleic Acids Research*, 43(7):e47, 2015.

[3] Gabriel E Hoffman and Eric E Schadt. variancePartition: interpreting drivers of variation in complex gene expression studies. *BMC Bioinformatics*, 17(1):483, 2016.

[4] Gabriel E Hoffman and Panos Roussos. dream: Powerful differential expression analysis for repeated measures designs. *Bioinformatics*, 2020.

[5] Keegan D Korthauer, Li-Fang Chu, Michael A Newton, Yuan Li, James Thomson, Ron Stewart, and Christina Kendziorski. A statistical approach for identifying differential distributions in single-cell RNA-seq experiments. *Genome Biology*, 17(1):222, 2016.

[6] Greg Finak, Andrew McDavid, Masanao Yajima, Jingyuan Deng, Vivian Gersuk, Alex K Shalek, Chloe K Slichter, Hannah W Miller, M Juliana McElrath, Martin Prlic, Peter S Linsley, and Raphael Gottardo. MAST: a flexible statistical framework for assessing transcriptional changes and characterizing heterogeneity in single-cell RNA sequencing data. *Genome Biology*, 16:278, 2015.

[7] F W Scholz and M A Stephens. K-Sample Anderson-Darling tests. *Journal of the American Statistical Association*, 82(399):918–924, 1987.

[8] Hyun Min Kang, Meena Subramaniam, Sasha Targ, Michelle Nguyen, Lenka Maliskova, Elizabeth McCarthy, Eunice Wan, Simon Wong, Lauren Byrnes, Cristina M Lanata, Rachel E Gate, Sara Mostafavi, Alexander Marson, Noah Zaitlen, Lindsey A Criswell, and Chun Jimmie Ye. Multiplexed droplet single-cell RNA-sequencing using natural genetic variation. *Nature Biotechnology*, 36(1):89–94, 2018.
